# Immunotherapy toxicity: identification and management

**DOI:** 10.1007/s10549-021-06480-5

**Published:** 2022-01-11

**Authors:** O. Gumusay, J. Callan, H. S. Rugo

**Affiliations:** University of California Helen Diller Family Comprehensive Cancer Center, San Francisco, CA USA

**Keywords:** Breast cancer, Immunotherapy, Immune checkpoint inhibitors, Toxicity, Immune-related adverse events, Retreatment

## Abstract

The widespread adoption of immunotherapy has revolutionized the treatment of various cancer types, including metastatic triple-negative breast cancer (TNBC), which has long been associated with poor prognostic outcomes. In particular, immune checkpoint inhibitors (ICIs) that target and inhibit programmed cell death-1 (PD-1) and programmed cell death ligand-1 (PD-L1), have shown promising results in the treatment of patients with metastatic TNBC. However, while manipulating the immune system to induce antitumor response, ICIs can also lead to a unique set of immune-related adverse events (IRAEs), which differ from standard chemotherapy toxicities due to their immune-based origin. These toxicities require highly specific management, including guidance from multidisciplinary specialists. The primary treatment strategy against IRAEs is systemic corticosteroid use, but additional treatment approaches may also involve supportive care, additional immunosuppression, and concurrent treatment delay or discontinuation. Given the rising prevalence of ICI therapy, it is essential to educate clinicians on the presentation and management of these potentially life-threatening events so that they are identified early and treated appropriately. Using data from recent clinical trials, this review will focus on known IRAEs, particularly those seen in patients with breast cancer, and will summarize their prevalence, severity, and outcomes. We will discuss optimal strategies for early recognition and management, as well as approaches toward cautious retreatment following resolution of IRAEs.

## Introduction

Immune checkpoint inhibitors (ICIs), which block inhibitory pathways that lead to tumor escape and facilitate immune response against tumor cells, have emerged as remarkable treatment options in oncology. ICIs provide antitumor activity by blocking intrinsic downregulators of immunity, such as cytotoxic T-lymphocyte antigen 4 (CTLA-4) and programmed cell death-1 (PD-1) or its ligand, programmed cell death ligand-1 (PD-L1) [1]. In recent years, the role of ICI therapy has been investigated in patients with breast cancer, which has changed the landscape of breast cancer treatment options for advanced triple-negative disease. Two agents have been approved as first line therapy in combination with chemotherapy for patients with PD-L1 + unresectable locally advanced and metastatic triple-negative breast cancer (TNBC): Atezolizumab, an antibody against PD-L1, and pembrolizumab, an antibody against anti-PD-1 [2–5]. Subsequent clinical trials have shown that the addition of pembrolizumab and atezolizumab to neoadjuvant chemotherapy increases pathologic complete response rates, regardless of PD-L1 status in patients with early-stage TNBC [6, 7]. Immune-related adverse events (IRAEs) most commonly involve the skin, endocrine glands, gastrointestinal system, and liver, but can affect almost any organ system. Other less frequent toxicities impact the cardiovascular, pulmonary, musculoskeletal, ocular, and central nervous systems [1, 8]. Compared to adverse events from chemotherapy, IRAEs typically present with delayed onset and prolonged duration. The majority of immune-related adverse events (IRAEs) are mild to moderate in severity and can be treated with appropriate immunosuppressive agents and/or immunomodulatory strategies in a timely manner; however, serious and rarely fatal adverse events can also occur. Awareness and management of these toxicities are mandatory in clinical practice. In this review, we briefly discuss the mechanisms of immune-related toxicity, then suggest methods for early diagnosis and effective management of the most common IRAEs from ICI therapy.

## Mechanism of toxicity

The exact pathophysiology underlying the occurrence of IRAEs is not clear. They are believed to be associated with the role that immune checkpoints play in maintaining immunologic homeostasis. Disinhibition of T-cell function by ICIs may lead to IRAEs. Translational studies have proposed that IRAEs may develop through a combination of pathways involving autoreactive T cells, autoantibodies and cytokines [1]. T-cell activation contributes to the development of IRAEs by leading to the production of inflammatory cytokines and B cell-mediated autoantibodies. There are differences in organ-specific toxicities in patients treated with anti-CTLA-4 therapy or anti-PD-1 therapy. Colitis and hypophysitis occur more frequently with anti-CTLA-4 therapy, whereas pneumonitis and thyroiditis appear to be more common with anti-PD-1 therapy [9–11]. Patients that develop thyroid disorders, while on anti-PD-1 therapy may have antithyroid antibodies, whether they existed at baseline or were detectable only after treatment initiation. One possible explanation of this phenomenon is that anti-PD-1 and anti-PD-L1 treatments, in addition to providing T-cell-mediated immunity, modulate humoral immunity and thereby enhance pre-existing antithyroid antibodies [12]. Studies have demonstrated that IRAEs may also develop through cross-reactive tumoral antigenicity, where the targeted T-cell antigens are present in both tumor and normal tissue [13]. For instance, a prospective study of autoimmune dermatologic toxicity in patients who received anti–PD-1 therapy for non-small cell lung cancer (NSCLC) describes T-cell antigens shared between tumor tissue and skin. Additionally, cytokines may play a role in the mechanism of IRAEs. Elevated levels of IL-17 have been observed in patients with ipilimumab-induced colitis in one clinical study and in preclinical models of colitis [14]. It is not fully understood why IRAEs occur in some patients but not others, but it may be that some patients have a predisposition to autoimmunity. Several studies have investigated the potential risk factors of IRAEs, including history of autoimmune disease, age, ethnicity, increased body mass index, genetic factors, and variations in the microbiologic composition of patients’ gastrointestinal flora [15]. While genetic variations could play a role in the risk of IRAEs, there was no clear evidence to support this [1]. Age does not appear to be a risk factor, as elderly patients tolerate ICIs much like younger patients [16]. Further studies are needed to confirm whether other epidemiologic factors contribute to the likelihood of developing IRAEs [17]. Toxicity differs depending on various therapy combinations. For instance, treatment-related adverse events (TRAEs), which are distinct from IRAEs, are observed more frequently when an ICI is combined with cytotoxic chemotherapy. However, IRAE rates are similar in patients treated with combination therapy as compared to those treated with ICI monotherapy. The only IRAE that appears to increase with combination therapy is pneumonitis, as the incidence of pneumonitis was higher in breast cancer patients treated with ICI and nab-paclitaxel than in those treated with monotherapy [18]. This could be the result of an overlapping toxicity effect, as both immunotherapy and taxanes have the potential to cause pneumonitis. Interestingly, rates of treatment discontinuation with the combination of chemotherapy plus immunotherapy are low compared to CTLA‐4 inhibitors alone or immunotherapy combinations [16].

## Immune checkpoint inhibitor-related toxicities

Onset of IRAEs generally occur within weeks to months of treatment initiation; however, they can develop at any time, even after discontinuation of ICI treatment [8]. IRAEs that arise during treatment with ICIs are termed as acute IRAEs, those that arise after completion of treatment as delayed IRAEs, and those that persist beyond 12 weeks of ICI discontinuation as chronic IRAEs. IRAEs that are likely to become chronic include endocrinopathies, arthritis, xerostomia, neurotoxicity and ocular events, whereas those that affect visceral organs, such as colitis, have much lower rates of becoming chronic [19]. These toxicities can affect almost any organ system and occur with a wide range of presentations, requiring multidisciplinary, collaborative management. As such, early recognition of symptoms and prompt intervention are important for effectively addressing these adverse events.

IRAEs are graded according to the Common Terminology Criteria for Adverse Events (CTCAE) from the US National Cancer Institute, which categorizes toxicity on a scale of 1 to 5, in ascending order of severity [20]. In the context of breast cancer in particular, the incidence of IRAEs in those treated with ICI therapy was reported in a meta-analysis including a total of 1746 patients with breast cancer across 27 studies: 34% of patients experienced IRAEs of any grade, and 15% experienced grades 3–4. With anti-PD-1 and anti-PD-L1 treatment specifically, IRAEs were reported in 28% and 53% of patients, respectively. Pembrolizumab (18%) and avelumab (10%) had significantly lower rates of IRAEs as compared to atezolizumab (74%) and nivolumab (81%) [19].

The frequency of IRAEs varies widely according to ICI agent and the organ-specific damage triggered, which may suggest that there is a specific population of individuals who are predisposed to IRAEs. There is no clear evidence explaining why some individuals have a greater tendency toward IRAEs than others, but it has been postulated that there could be a genetic explanation. For instance, some individuals might have a predisposition to autoimmunity [16]. Several studies have suggested an increased risk of IRAEs in patients with pre-existing autoimmune diseases. Previous studies demonstrated that about 10% of patients who developed rheumatic IRAEs had a family history of autoimmune disorders. Genetic variations and the composition of host microbiota could also play a role in the risk of IRAEs [1, 16]. Even so, the role of genetics in determining one’s likelihood of developing IRAEs is not well understood.

### Endocrine toxicity

Endocrine toxicities associated with ICI therapy include hypothyroidism or hyperthyroidism, thyroiditis, hypophysitis, primary adrenal insufficiency, and insulin-dependent diabetes mellitus. The time to onset of endocrine IRAEs varies by type of agent and endocrinopathy [21]. Unlike other IRAEs, which resolve with treatment, endocrine toxicities are almost always permanent and require lifelong hormone replacement therapy [22].

#### Thyroiditis

**Fig. 1:**
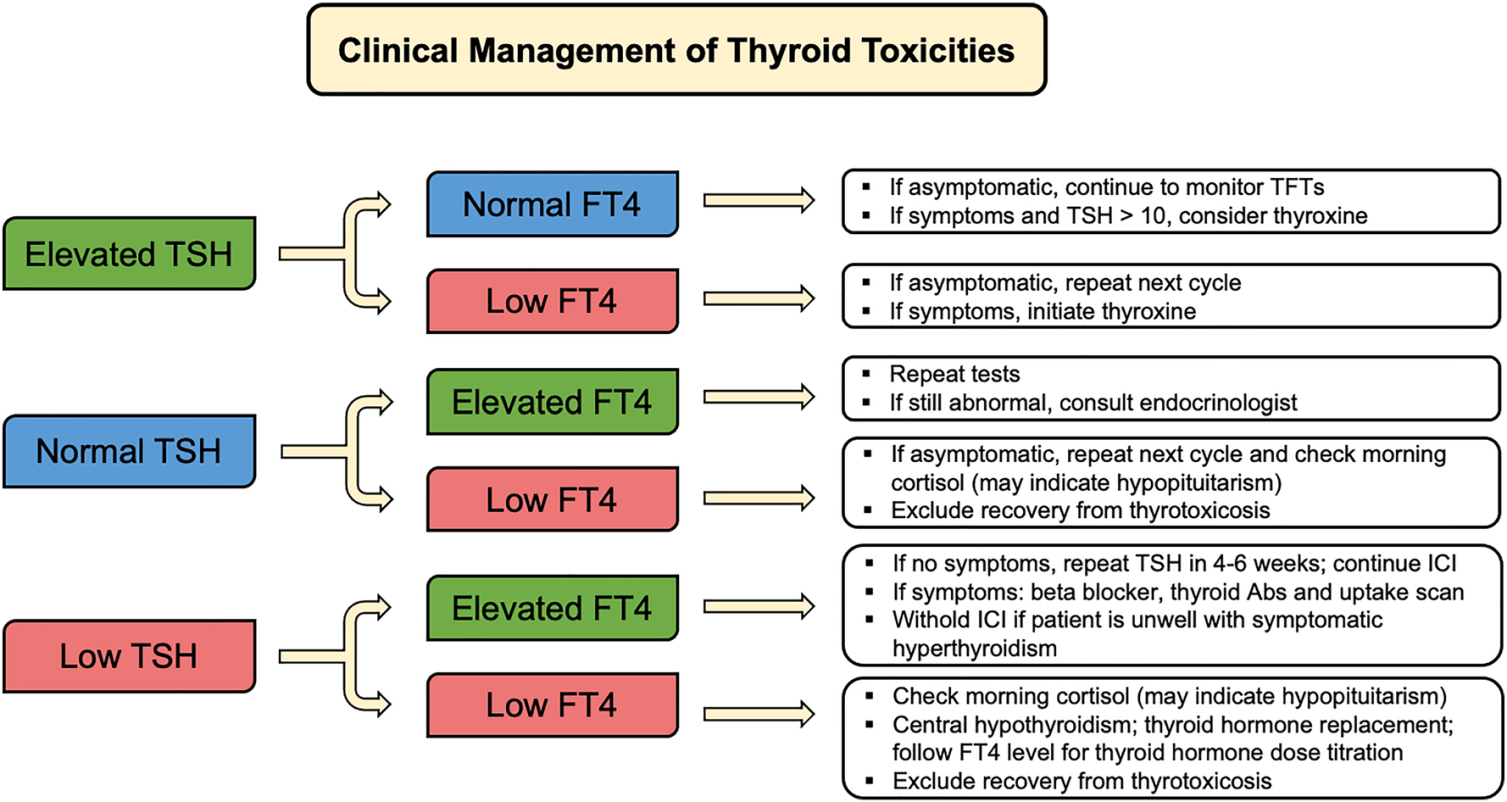
Flow diagram for monitoring and management of immune-related thyroid toxicities in patients treated with ICIs. *Abs* antibodies; *FT4* free thyroxine; *ICI* immune checkpoint inhibitor; *TSH* thyroid-stimulating hormone

The pathogenesis of thyroid disorders following ICIs is not well understood. It is thought to be mediated by T cells rather than B cell autoimmunity [23]. Hypothyroidism is more common than hyperthyroidism in patients treated with ICI therapy. The median reported time to new onset or exacerbation of pre-existing thyroid dysfunction is typically 4–7 weeks after initiation of ICI [24]. Hyperthyroidism is often transient and may precede hypothyroidism [21]. In clinical trials evaluating ICI therapy in patients with breast cancer, the incidence of hypothyroidism of any grade was 4% to 18.0%, whereas the reported rate of hyperthyroidism varied from < 1% to 9.8% (Table 1) [4, 25, 26]. Most thyroid toxicities found in breast cancer patients are reported as grades 1–2, often asymptomatic and detected by routine blood tests (TSH and FT4). Investigation of hypothyroidism should differentiate primary hypothyroidism (in which TSH levels are high and T4 levels are low) from secondary hypothyroidism (characterized by low TSH and T4 levels and hypophysitis or pituitary dysfunction) (Fig. 1). Monitoring, presentation and diagnosis of thyroid disorders following ICIs is provided in Table 2. For patients with primary hypothyroidism, additional testing for thyroid antibodies, including thyroid peroxidase (TPO) antibody, is required. Blood tests such as TSH and free T4 should be carried out at baseline and before every infusion, or at least every 4 to 6 weeks during ICI. This interval can be extended to every 12 to 18 weeks in patients who have normal thyroid function or who experience no symptoms. Patients with asymptomatic and subclinical hypothyroidism who have elevated TSH with normal free T4 can be proceed with ICI, but should be monitored routinely. Levothyroxine can be initiated for TSH levels above 10 mIU/L. Hyperthyroidism can occur during the thyrotoxic phase of thyroiditis, or from other causes of thyrotoxicosis, including Graves disease (defined as high free T4 or total T3 with low or normal TSH). Most commonly, patients with thyroiditis are asymptomatic. Thyroiditis is a self-limiting toxicity that can cause permanent hypothyroidism after the thyrotoxic phase. Thyrotoxicosis is diagnosed with low or suppressed TSH (< 0.01 mIU/L), high free T4 and/or total triiodothyronine (T3), and may be symptomatic in the setting of high T4. Presentation of thyrotoxicosis includes weight loss, palpitations, heat intolerance, tremors, anxiety, and diarrhea. If patients are symptomatic, propranolol to manage symptoms until resolution should be considered with endocrine consultation. Testing for TSH receptor antibodies is required when there are clinical features of or suspicion for Graves disease, such as ophthalmopathy. (Table 2) [8, 22].

**Table 1:** Common IRAEs in patients with breast cancer

Trial	Agent	Ph	#	Hypothyroidism (%)		Hyperthyroidism (%)		AI (%)^a^		Dermatologic (%)		Hepatitis (%)		Colitis/Diarrhea (%)		Pneumonitis (%)		IRR (%)	
				Any Gr	Gr ≥ 3	Any Gr	Gr ≥ 3	Any Gr	Gr ≥ 3	Any Gr	Gr ≥ 3	Any Gr	Gr ≥ 3	Any Gr	Gr ≥ 3	Any Gr	Gr ≥ 3	Any Gr	Gr ≥ 3
Trials of immunotherapy in neoadjuvant setting																			
KEYNOTE-522^c^ [6]	Pembro + CT	III	781	13.7	< 1	4.6	< 1	4.1	2.3	4.4	3.8	1.4	1.2	1.7	< 1	1.3	< 1	16.9	2.6
I-SPY2 [28]	Pembro + CT	II	69	10.1	1.4	5.8	NR	8.7	7.2	31.9	NR	2.9	2.9	1.4	1.4	4.3	NR	NR	NR
IMpassion031[7]	Atezo + CT	III	164	7	NR	3	NR	NR	NR	49	4	1	NR	1	1	NR	NR	10	1
GeparNuevo [42]	Durva + CT	II-R	92	7.6	NR	9.8	NR	1.1	NR	14.1	NR	8.7	1.1	NR	NR	1.1	NR	3.3	NR
Trials of immunotherapy in advanced and/or metastatic setting																			
KEYNOTE-012 [2]	Pembro	Ib	32	NR	NR	NR	NR	NR	NR	6.3	NR	6.3^b^	NR	12.5	NR	NR	NR	NR	NR
KEYNOTE-086 Coh A [31]	Pembro	II	170	11.8	NR	5.3	NR	NR	NR	6.5	NR	NR	NR	1.2	NR	4.1	< 1	1.8	NR
KEYNOTE-086 Coh B [38]	Pembro	II	84	9.5	NR	4.8	NR	1.2	NR	1.2	1.2	NR	NR	1.2	NR	2.4	NR	1.2	NR
KEYNOTE-119 [44]	Pembro	III	309	7.8	< 1	4	NR	1	< 1	1.6	1	NR	NR	< 1	NR	1.9	< 1	NR	NR
KEYNOTE-355 [5]	Pembro + CT	III	562	15	< 1	5	< 1	NR	NR	2	2	NR	NR	2	< 1	2	1	4	1
NCT01375842 [25]	Atezo	I	116	4	NR	NR	NR	1	NR	22	NR	8	NR	10	1	1	NR	NR	NR
IMpassion130 [4, 26]	Atezo + CT	III	451	18.0	NR	5	< 1	< 1	< 1	34	< 1	15.3^d^	5.1	1	< 1	4.0	< 1	1.1	NR
IMpassion131 [37]	Atezo + CT	III	431	12.8	NR	5.1	NR	< 1	NR	32	< 1	1.6	< 1	< 1	< 1	3.7	< 1	3.2	< 1
JAVELIN [45]	Avelumab	Ib	168	4.8	NR	< 1	NR	NR	NR	< 1	NR	1.8	1.8	NR	NR	1.8	< 1	14.3	NR

*#* number of patients; *AI* adrenal insufficiency; *Atezo* atezolizumab; *Coh* cohort; *Durva* durvalumab; *Gr* grade; *IRR* infusion-related reactions; *NR* not reported; *Pembro* pembrolizumab; *Ph* phase

^**a**^Rate of adrenal insufficiency (primary and secondary)

^b^Elevated liver enzymes (AST and ALT)

^c^Adverse events during the neoadjuvant phase at second interim analysis

^d^Hepatitis with diagnoses and lab abnormalities. Diagnoses of any grade: 2.2%, grade 3–4: 1.3%. Lab abnormalities of any grade: 13.7%, grade 3–4: 3.8%

**Table 2:** Monitoring, presentation and diagnosis of common IRAEs

Toxicity	Monitoring	Presentation	Diagnosis
Endocrine			
Thyroid	TSH & free T4 at baseline and every 4–6 weeks during ICI, with follow up every 12 weeks thereafter, as indicated	Hypothyroidism Hyperthyroidism Myxedema Thyroid storm	TSH, free T4 Morning cortisol level for concurrent adrenal insufficiency TSH receptor antibodies if Graves disease is suspected
Adrenal insufficiency	TSH & free T4 at baseline and every 4–6 week on ICI Consider baseline ACTH and cortisol in high risk patients	Hypophysitis Dysfunction of thyroid, adrenal or gonadal axis Symptoms: Headache, dizziness, nausea/ emesis, anorexia, fatigue	Lab: Evaluate morning cortisol and ACTH TSH, free T4 LH, FSH, testosterone (men), estradiol (women) Imaging: MRI of sella Secondary adrenal insufficiency: Low ACTH, low cortisol Central hypothyroidism: Low TSH, low free T4
Non-endocrine			
Dermatologic	Complete skin and mucous membrane examination Review prior immune-related skin disorders	Rash, pruritus, bullous dermatitis Dermatomyositis Dermal hypersensitivity reaction Sweet syndrome Pyoderma gangrenosum DRESS SJS/TEN	Rule out alternative etiology of skin problem (e.g., infection, other drug rash, skin lesion linked to another systemic disorder) Complete skin examination and determination of lesion type Consider skin biopsy
Hepatitis	Comprehensive metabolic panel (serum transaminase and bilirubin) at baseline Repeat every 2–3 weeks during ICI	Elevation of AST/ ALT Fulminant hepatitis	Comprehensive metabolic panel Rule out infectious causes: viral studies Rule out drug-induced hepatitis, including alcohol ANA, ANCA, ASMA if autoimmune hepatitis suspected Rule out NASH and thrombosis Abdomen CT to evaluate liver metastases Consider liver biopsy
Diarrhea or colitis	Evaluate baseline bowel habits Follow up any GI symptoms and signs	Diarrhea Urgency Abdominal pain and/or cramping Fever	Evaluate baseline bowel habits CBC, Comprehensive metabolic panel, ESR, TSH, CRP Rule out infection: stool culture, Clostridium difficile, CMV DNA PCR, stool ova and parasites Consider lactoferrin/calprotectin CT Abdomen/pelvis Consider GI consultation for EGD/colonoscopy with biopsy
Pneumonitis	Follow up any respiratory symptoms and signs	Cough, chest pain, wheezing, shortness of breath, new hypoxia, fatigue, respiratory failure	Consider CT Chest Consider infectious work-up: nasal swab, sputum culture Consider bronchoscopy with BAL and consider transbronchial lung biopsy •Imaging findings are variable and include cryptogenic organizing pneumonia, non-specific interstitial pneumonitis, hypersensitivity pneumonitis, or usual interstitial pneumonitis/pulmonary fibrosis

*ACTH* adrenocorticotropic hormone; *ANA* anti-nuclear antibodies; *ANCA* anti-neutrophil cytoplasmic antibody; *ASMA* anti-smooth muscle antibody; *BAL* bronchoalveolar lavage; *CMV* cytomegalovirus; *CRP* C-reactive protein; *CT* computed tomography; *DRESS* drug rash with eosinophilia and systemic symptoms; *EGD* esophagogastroduodenoscopy; *ESR* erythrocyte sedimentation rate; *FSH* follicle-stimulating hormone; *GI* gastrointestinal; *ICI* immune checkpoint inhibitor; *LH* luteinizing hormone; *MRI* magnetic resonance imaging; *NASH* non-alcoholic steatohepatitis; *PCR* polymerase chain reaction; *SJS/TEN* Stevens-Johnson syndrome or toxic epidermal necrolysis; *T4* thyroxine; *TSH* thyroid-stimulating hormone

#### Adrenal insufficiency

Adrenal insufficiency can be seen in cancer patients treated with anti-PD-1 and anti-PD-L1 therapy [27]. Adrenal insufficiency can present as either primary insufficiency or, more commonly, secondary insufficiency referred to as hypophysitis. Symptoms of secondary adrenal insufficiency can be non-specific. Acute symptoms may include headache, photophobia, dizziness, nausea/emesis, fevers, anorexia, visual field cuts or severe fatigue, while chronic symptoms can include fatigue and weight loss. As hypophysitis can affect the thyroid, adrenal or gonadal axes, the following tests should be performed for diagnosis: ACTH, cortisol (AM), follicle-stimulating hormone (FSH), luteinizing hormone (LH), TSH, FT4, electrolytes, testosterone in men, and estrogen in premenopausal women [28]. Low levels of ACTH, AM cortisol, sodium, potassium, testosterone and DHEA-S are indicative of hypophysitis. Brain MR with pituitary/sellar cuts may be performed in patients with multiple endocrine abnormalities with or without new severe headaches or complaint of vision change [22]. Work-up for primary adrenal insufficiency should include serum cortisol, comprehensive metabolic panel and renin levels. If abnormal levels are detected, ACTH, LH, FSH, and testosterone should be evaluated. Primary and secondary adrenal sufficiency can be distinguished by checking the levels of cortisol and ACTH. Hallmarks of primary adrenal insufficiency include low morning cortisol (< 5) with ACTH above the reference range, and abnormal cosyntropin stimulation test with or without abnormal electrolytes and symptoms. Hypotension, orthostatic hypotension, low sodium and high potassium can also be found. However, patients who develop adrenal insufficiency secondary to ICI-induced hypophysitis have low levels of ACTH and cortisol [22]. It is critical to check morning cortisol in patients who are undergoing surgery on ICI because surgery can bring out symptomatic adrenal insufficiency, which could be life-threatening.

Adrenal insufficiency was observed in 8.7% of breast cancer patients who received pembrolizumab in the I-SPY2 trial. Three of these cases were classified as hypophysitis and one as primary adrenal insufficiency. The remaining two patients were not evaluated due to initiation of steroid therapy prior to work-up [29]. The FDA Oncology Drug Advisory Council met on February 9^th^, 2021 to review data from KEYNOTE-522. In this interim analysis, 2.2% of patients experienced primary adrenal insufficiency, and 1.9% of patients experienced hypophysitis. One patient died from adrenal crisis on the day following her breast surgery. Her cortisol level was only 3 nmol/L (normal range 172–497), likely representing undiagnosed adrenal insufficiency at the time of surgery [30].

#### Diabetes mellitus

De novo diabetes mellitus (DM) with ICI therapy occurs at low frequency and can be either type 1 or type 2. Blood glucose levels should be regularly monitored in patients treated with ICIs to detect this. Patients with new-onset polyuria, polydipsia, weight loss, nausea and/or vomiting should be evaluated for possible development of type 1 DM. Fasting glucose is the preferred diagnostic test for suspected new-onset hyperglycemia. The role of steroids to prevent loss of beta cells in the islands of the pancreas is unclear and steroids can negatively affect blood glucose levels. In the KEYNOTE-522 trial, which enrolled patients with early-stage breast cancer to evaluate the addition of pembrolizumab to neoadjuvant chemotherapy, type 1 DM was reported in 2 patients (0.3%). Two patients (1.2%) were diagnosed with type 1 DM with single agent pembrolizumab treatment [31], and 1 patient experience immune-related DM with atezolizumab and nab-paclitaxel combination therapy in the metastatic setting [4].

### Non-endocrine toxicities

#### Dermatologic toxicity

Dermatologic toxicity is common in patients treated with PD-1 or PD-L1 blockade and generally develops early in the course of treatment, within days or weeks, although delayed onset can occur. Severe skin adverse events are rare. Dermatologic toxicities of any grade are reported to occur in < 1% to 49% of patients with breast cancer receiving ICI treatment, though the majority of dermatologic toxicity is reported as grades 1 or 2 (Table 1). The presentation of dermatologic IRAEs is diverse. Those most commonly reported are maculopapular rash and pruritus [32]. Although less frequent, psoriasis and lichenoid, eczematous, and bullous dermatitis have also been reported in patients treated with ICI [33]. The first step when ICI-treated patients present with skin AEs should be to rule out any other etiologic factors that could be contributing to the skin condition, such as an infection, a drug-induced lesion, or a skin condition linked to another systemic disorder. Next, the grade of dermatologic toxicity and the general patient status must be evaluated. Blood tests, including blood cell count, liver tests and kidney tests, will help evaluate for dermatological emergencies, such as drug rash with eosinophilia and systemic symptoms (DRESS), acute febrile neutrophilic dermatosis (Sweet syndrome), Stevens-Johnson syndrome, or toxic epidermal necrolysis (TEN) [8, 22, 34].

#### Immune-related hepatitis

Hepatitis occurs in 1%-6.3% of patients with breast cancer receiving ICI therapy (Table 1). Immune-related hepatitis is typically mild, but can be severe or even fatal in rare cases. The most common presentation of immune-related hepatitis is asymptomatic with aspartate transaminase (AST) and alanine transaminase (ALT) abnormalities, with or without hyperbilirubinemia. Median onset of transaminase elevation is approximately 3–9 weeks after starting ICI therapy [24]. Serum transaminases and bilirubin should be measured prior to initiation of ICIs and before every cycle of ICI treatment. The differential diagnosis of transaminase elevation during ICI treatment includes disease-related causes, concomitant drug intake including alcohol, and infectious causes, especially viral hepatitis. However, initiation of therapy for IRAEs should not be delayed while pending serological results if there is no other obvious cause. Liver biopsy can be considered to assess more severe hepatic reactions, such as transaminase levels greater than five times the upper limit of normal (Table 2) [8, 22].

#### Gastrointestinal toxicity

Gastrointestinal (GI) toxicity may present with symptoms of colitis, including watery diarrhea, cramping, and urgency. Diarrhea has an incidence of ~ 20% in patients treated with anti-PD-1 therapy; however, colitis with evidence of colon inflammation is only reported in 1% of such patients. Colitis is more commonly seen with anti-CTLA-4 monotherapy than with anti-PD-1/PD-L1 inhibitors [35]. Anti-CTLA-4 drugs, such as ipilimumab and tremelimumab, have not been approved in the treatment of patients with breast cancer, but there are many ongoing trials investigating the therapeutic role of ipilimumab in breast cancer. In addition to diarrhea, the presence of abdominal pain, rectal bleeding, mucus in the stool, and fever should raise suspicion for colitis, which is a potentially life-threatening complication of ICI treatment. The investigation of potential infectious causes should be performed in diagnosing ICI-related colitis. CT exams should also be performed to evaluate the extent and severity of colitis and to rule out bowel perforation. Endoscopy, while not routine for mild cases, can help diagnose patients with refractory or recurrent colitis and distinguish from cytomegalovirus associated colitis [32]. Colonic biopsy findings of colitis typically reveal a mixed lymphocytic and neutrophilic infiltrate with apoptotic mucosal epithelial cells and crypt abscess [36]. Associated diffuse enteritis can be seen simultaneously in patients with or without colitis. Upper GI symptoms, such as dysphagia and epigastric pain, have been reported. Endoscopic lesions outside the colon, including esophageal ulcerations, gastritis and duodenitis, can also occur. Stool culture for bacterial enteropathogens and stool analyses for Clostridium difficile toxin should be obtained to rule out infectious etiology. Diarrhea and colitis have been reported in < 1% to 12.5% of patients with breast cancer treated with ICI (Table 1). The majority of these cases are low grade and manageable with appropriate care [2, 37, 38]. Diarrhea and/or colitis may recur months after discontinuation of ICI therapy, and cases like these must be distinguished from inflammatory bowel disease [39].

#### Pneumonitis

Pneumonitis is the most common pulmonary toxicity of ICI therapy and has variable onset, clinical presentation, and imaging findings [8]. The overall incidence of pneumonitis is low with reported rates of 1–4.1% in patients with breast cancer (Table 1) [25, 31]. However, it is potentially life-threatening and should be assessed by CT in any patient who develops new respiratory symptoms, such as new cough, shortness of breath or hypoxia, chest pain, fatigue with activities of daily living (ADL), and new or increasing requirement for supplementary oxygen during ICI treatment. In one case, a breast cancer patient with malignant pleural effusion presented with dry cough, dyspnea, and fever after receiving pembrolizumab treatment. A chest CT showed ground glass opacity, and a transbronchial lung biopsy revealed focal septal lymphocytic infiltration. The patient recovered with 1.0 mg/kg/day steroid therapy [40].

In general, as pulmonary symptoms and signs can sometimes suggest disease progression, particularly in the context of lung metastases, any new respiratory symptom should be evaluated carefully. Some asymptomatic patients are diagnosed incidentally on imaging studies [10]. Imaging findings are variable and can include cryptogenic organizing pneumonia, non-specific interstitial pneumonitis, hypersensitivity pneumonitis, or usual interstitial pneumonitis/pulmonary fibrosis [8]. Pre-existing pulmonary fibrosis or chronic obstructive pulmonary disease can delay the diagnosis of immune-related pneumonitis. Acute interstitial pneumonitis/diffuse alveolar damage syndrome is the most acute life-threatening event. Even though pneumonitis can be observed at any time, patients commonly experience pneumonitis months after treatment initiation, which is later than other IRAEs. In general, lung biopsy is not required for management of patients, but can be considered if there is any suspicion of acute infection or lymphangitic spread of lung metastases. Alternatively, bronchoscopy with bronchoalveolar lavage is sometimes recommended by a pulmonologist for symptomatic pneumonia and can be helpful to identify infections, including potential opportunistic or atypical agents (Table 2).

### Rare immune-related adverse events

#### Neurologic toxicity

Neurologic IRAEs occur rarely in patients receiving ICI, but can cause substantial morbidity if not recognized and treated early. Neurologic IRAEs are reported to occur within 4.5 weeks of initiation of anti-PD-1 therapy [41]. A range of neurological events have been described in patients with breast cancer, including headache, encephalitis, aseptic meningitis, peripheral neuropathy, adverse events affecting cranial nerves, myasthenia gravis, and Guillain-Barre like syndrome (GBS)[2, 4, 6, 7, 25, 42]. Additionally, central neuropathy and transverse myelitis were reported with previous ICI trials in patients with variable solid tumors. The most commonly reported neurologic toxicities were headache, encephalopathy, meningitis and Myasthenic syndrome, and a majority of these events were low grade [41]. Previous trials have reported severe neurologic IRAEs resulting in fatality, such as encephalitis and myasthenia gravis. The presentation of neurologic IRAEs can be diverse with non-specific symptoms. Peripheral neuropathies have been reported with ICIs in which nerve conduction studies are helpful. Patients with myasthenia gravis are presented with progressive or fluctuating muscle weakness, generally proximal to distal. Bulbar involvement and/or respiratory muscle weakness are features of ICI-related myasthenia gravis. Patients presenting with headaches, neck stiffness and photophobia during ICI therapy should be investigated for aseptic meningitis. In addition to these symptoms described, patients who present with altered mental status and/or seizures should be evaluated for potential encephalitis. It is necessary to rule out other potential factors, such as progression of breast cancer, central nervous system (CNS) metastasis or leptomeningeal spread, paraneoplastic syndrome, vitamin B12 deficiency, diabetic neuropathy, seizure activity, infection and metabolic derangement. Recommended assessments include brain MR, spine MR, lumbar puncture, and electroencephalography (EEG). Appropriate laboratory tests should be performed depending on the potential causes of neurologic toxicity. Neurology consultation should be considered if there is suspicion for severe neurologic toxicities such as myasthenia gravis, Guillain–Barre syndrome (GBS), aseptic meningitis, encephalitis or transverse myelitis [8, 22, 43].

#### Cardiovascular toxicity

IRAEs of the cardiovascular system are rare, but potentially permanent and/or fatal. A range of cardiac events have been reported after treatment with ICIs, including myocarditis, pericarditis, arrhythmias, cardiomyopathy and impaired ventricular function, acute heart failure and cardiac arrythmia. Myocarditis is one of the most common cardiovascular toxicities and occurs within 4 weeks of ICI therapy initiation. In some patients with cardiac IRAEs, severe adverse events, such as myositis and myasthenia gravis, were reported concurrently with myocarditis. Clinical presentation of cardiac IRAEs can include fatigue, weakness, dyspnea, chest pain, palpitations or symptoms of congestive heart failure, depending on the type of cardiac dysfunction [8]. Electrocardiography and cardiac serum biomarkers including creatinine kinase and troponin levels should be checked to investigate cardiac toxicity. Additionally, echocardiography to assess left ventricular fraction and cardiac MR with gadolinium enhancement to assess inflammation secondary to myocarditis are useful diagnostic tools [22]. Endomyocardial biopsy is the gold standard for the diagnosis; however, it is rarely performed as the first step in diagnosis due to its invasiveness [44]. Additionally, it has low sensitivity and high interobserver variability in interpretation of biopsy samples [45]. For these reasons, endomyocardial biopsies are only recommended for uncommon presentations of myocarditis [46].

#### Rheumatologic toxicity

Identifying rheumatologic IRAEs in patients with cancer is challenging due to the difficulty in distinguishing these IRAEs from other musculoskeletal complaints, as this patient population has a high baseline frequency of musculoskeletal symptoms. Myalgias, arthralgias, inflammatory arthritis and polymyalgia rheumatica are the most common IRAEs. Joint examination, functional assessment of joints, imaging modalities, and occasionally laboratory tests can be helpful for diagnosis. Variable presentations of rheumatologic IRAEs have been reported, including sicca syndrome, myositis, giant cell arthritis, systemic lupus erythematosus, and sarcoidosis. Rarely, severe myositis presenting with muscle weakness and elevated creatine kinase (CK) can be fatal and has been reported more commonly in patients receiving PD-1/ PD-L1 inhibitors. Rheumatologic IRAEs such as arthralgia, myalgia, pain in extremities, back pain, dry eye, xerostomia, elevated rheumatoid factor, anti-nuclear antibody (ANA) positivity and myositis have been reported in patients with breast cancer receiving ICI treatment [2, 6, 47, 48]. Early rheumatology consultation and the introduction of disease-modifying drugs beyond steroids can help optimize treatment and prevent irreversible joint damage [8, 49].

#### Renal toxicity

Renal IRAEs are rare in patients with breast cancer treated with ICI therapy. Recent studies suggested that the incidence of IRAEs may be under-reported due to mild symptoms and signs [50]. Presentation varies and can include elevated serum creatinine, electrolyte imbalance, altered urinary output and worsening hypertension. Nephritis, hyponatremia, hypertension and hypokalemia have been reported in previous clinical trials involving breast cancer patients treated with ICIs. The differential diagnosis for patients who develop renal dysfunction on ICIs includes infection, dehydration, nephrotoxic drugs, urinary tract obstruction, contrast agents. Serum sodium, potassium, creatinine and urea should be measured before every infusion of ICI therapy. In addition, urinary protein followed by autoimmune markers, such as anti-nuclear antibody, anti-neutrophil cytoplasmic antibody, rheumatoid factor, anti-double-stranded DNA antibodies and serum complement levels, might be helpful toward understanding the underlying pathological process [22]. Clinical findings and laboratory tests are not ideal in diagnosing underlying renal lesions, so kidney biopsies are necessary to definitively diagnose the type of renal damage. Acute interstitial nephritis, minimal change disease, lupus-like disease and acute thrombotic microangiopathy have been reported as pathologic findings with ICI treatment [50, 51].

#### Ocular toxicity

Variable ocular IRAEs occur depending on the affected area of the eye. These can include retinal choroidal disease, optic neuropathy, or various presentations of ocular inflammation, such as peripheral ulcerative keratitis, uveitis, episcleritis, blepharitis, orbital inflammation and orbitopathy (idiopathic or thyroid-induced orbitopathy) [52]. The most commonly reported IRAEs are dry eye and uveitis. Patients can present with blurred or distorted vision, changes in color vision, blind spots, photophobia, eye pain, eyelid swelling, and proptosis. Episcleritis can be associated with red or purple discoloration of the eye, and uveitis may present with eye redness. Ocular toxicity has been reported concurrently with extraocular IRAEs, particularly colitis [52, 53]. While oncologists can perform penlight inspection or examine patients for visual acuity and color vision, referral to ophthalmology is recommended for all those patients who experience visual symptoms with ICI therapy. In patients with breast cancer, dry eye, uveitis, and ocular inflammatory toxicities were reported during ICI therapy in clinical trials. In one case of a patient with mTNBC, acute macular neuroretinopathy was reported after receiving atezolizumab [54].

#### Hematologic toxicities

Hematological IRAEs have been reported in patients treated with ICI, although rarely. Examples include neutropenia, thrombocytopenia, myelodysplasia, aplastic anemia, autoimmune hemolytic anemia and immune thrombocytopenic purpura. Cytopenia that is persistent or progressive after receiving ICI therapy should be investigated for autoimmune causes. The differential diagnosis for cytopenia includes cancer progression, bone marrow involvement, gastrointestinal bleeding and drug effect [55]. As the optimal management of hematological toxicities is largely unknown, prompt consultation with a hematologist is recommended to initiate high dose corticosteroid and/or other immunosuppressive drugs [8].

## Infusion-related reactions

During or following infusion with ICIs, patients can present with constitutional symptoms such as fever, rigor, pruritus, hypotension, dyspnea, chest comfort, rash, urticaria, angioedema, wheezing or tachycardia, and rarely anaphylaxis requiring urgent intervention. In the literature, infusion reactions have been reported most commonly with avelumab. In the JAVELIN trial, for instance, 14.3% of patients with breast cancer experienced infusion reactions of any grade after treatment with avelumab [48]. The rates of all grades infusion-related reactions range from 1.1 to 16.9% in patients with breast cancer receiving ICI (Table 1) [4, 6]. Most infusion reactions are mild and present with low-grade fever, chills, headache or nausea; however, severe reactions of grades 3–4 have been reported in < 1% to 2.6% of patients with breast cancer in clinical trials (Table 1). Mild to moderate reactions are managed with a variety of methods, including symptomatic treatment, reducing the rate of infusions, temporarily interrupting infusions, or adding premedication for subsequent infusions, while severe reactions should be managed urgently with anti-histamines, oxygen, fluids, opioids, corticosteroids and bronchodilators. Permanent discontinuation of the ICI is recommended when grade 3–4 infusion reactions occur [8, 22].

## Management of immunotherapy-related toxicities

ICB-related toxicities result from excessive host immune response directed against normal organs. As such, most IRAEs can be treated by inducing temporary immunosuppression with oral corticoids, high dose steroid therapy (oral prednisolone 1–2 mg/kg/day or IV equivalent), or additional immunosuppressants in more severe cases. Proton pump inhibitor or H2 blockers for gastrointestinal prophylaxis can be considered with high dose steroid treatment. For steroid-refractory cases, including patients with severe IRAEs that are not responsive to steroids within 48–72 h, early initiation of additional immunosuppressants or plasmapheresis can be considered with close guidance from a disease-specific subspecialist. Examples of these immunomodulatory agents include infliximab, tumor necrosis factor inhibitors (TNFi), mycophenolate mofetil, anti-thymocyte globulin (ATG), calcineurin inhibitors, methotrexate, or intravenous gammaglobulin (IVIG). Guidance on prescribing routine prophylactic antibiotics to reduce the potential opportunistic infection in patients receiving steroids remains unclear. For patients receiving at least 20 mg prednisone or equivalent/day for ≥ 4 weeks, the addition of prophylactic antibiotics for pneumocystis pneumonia (PCP) can be considered [8, 22]. Prophylaxis against fungal infections (e.g., fluconazole) is recommended in patients receiving prednisone ≥ 20 mg daily for ≥ 6–8 weeks. The management of immunotherapy toxicities should depend on grade of severity, type, and number of adverse events. Patient’s medical history, comorbidities, underlying disease status, and ability to tolerate corticosteroids must be incorporated into decisions about optimal treatment strategies. The management of IRAEs is summarized in Table 3.

**Table 3:** Classification and management of common and rare IRAEs

Toxicity	CTCAE-V5 classification [19]	Management [17, 21]
Common endocrine IRAEs		
Thyroid	Grade 1: Asymptomatic, clinical or diagnostic observations only Grade 2: Moderate symptoms, medical intervention indicated Grade 3: Severe symptoms; limiting self-care ADL; medical intervention or hospitalization required Grade 4: Life-threatening consequences; urgent intervention indicated	Asymptomatic hypothyroidism: Thyroid hormone replacement if TSH > 10 mIU/L Symptomatic hypothyroidism: Thyroid hormone replacement Hyperthyroidism: If symptomatic, consider endocrine consultation and propranolol for symptom control ICI can be continued in the setting of hypothyroidism or thyrotoxicosis
Adrenal Insufficiency	Grade 1: Asymptomatic or mild, clinical or diagnostic observations only Grade 2: Moderate symptoms, minimal, local or non-invasive intervention indicated Grade 3: Severe or medically significant but not immediately life-threatening; hospitalization indicated; limiting self-care ADL Grade 4: Life-threatening consequences; urgent intervention indicated	Endocrine consultation Hold ICI until acute symptoms resolve, and hormone replacements are initiated Treat with hormone replacement as indicated: •Secondary adrenal insufficiency: steroid replacement (physiologic steroid replacement hydrocortisone 20 mg AM, 10 mg PM). Acutely symptomatic or hospitalized patients may require stress doss hydrocortisone (e.g., 50 mg every 6–8 h) •Central hypothyroidism: Thyroid hormone replacement and follow free T4 level for dose titration
Common non-endocrine IRAEs		
Dermatologic	Grade 1: Macules/papules covering < 10% the BSA with or without symptoms (pruritus, burning, tightness) Grade 2: Macules/papules covering 10–30% BSA with or without symptoms (pruritus, burning, tightness); limiting instrumental ADL Grade 3: Macules/papules covering > 30% BSA with or without associated symptoms: Limiting self-care ADL Grade 4: Papulopustular rash associated with life-threatening superinfection; SJS/TEN, bullous dermatitis covering > 30% of BSA and requiring ICU admission	Grade 1: Emollients, topical corticosteroids and/or oral or topical anti-histamines. Avoid skin irritants, avoid sun exposure. Proceed with ICI treatment Grade 2: High potency topical corticosteroids, and/or oral steroids. Proceed with ICI Grade 3–4: Withhold ICI. Treat with systemic 1–2 mg/kg/d steroids and dermatology consultation. Treat until symptoms improve to grade ≤ 1 then taper over 4–6 weeks •Consider IVIG or rituximab for grade $\ge$ 3 bullous dermatitis •Permanently discontinue ICI treatment if grade 4 or SJS/TEN •Consider gabapentin/pregabalin for grade $\ge 3$ pruritus
Hepatitis	Grade 1: ALT or AST > ULN-3× ULN Grade 2: ALT or AST 3–5× ULN Grade 3: 5–20× ULN Grade 4: > 20× ULN	Grade 1: Continue ICI treatment with increased frequency of LFT monitoring Grade 2: Hold ICI until recovery to grade ≤ 1; start systemic 0.5-1 mg/kg/d if no improvement Grade 3–4: Hold ICI; monitor liver enzymes, hepatology consult; start 1–2 mg/kg/d steroids Grade 4: Permanently discontinue ICI; start 1–2 mg/kg/d steroids Grade > 1 transaminitis with elevated bilirubin: •Bilirubin 1–2× ULN: hold ICI, initiate 1–2 mg/kg/d steroids •Bilirubin 3–4× ULN: permanently discontinue ICI; initiate 1–2 mg/kg/d steroids For steroid-refractory cases, or if no improvement after 3 days, consider mycophenolate mofetil and cyclosporine; infliximab is contraindicated because of concerns about hepatotoxicity
Diarrhea	Grade 1: Increase of < 4 stools per day over baseline Grade 2: Increase of 4–6 stools per day over baseline; limiting instrumental ADL Grade 3: Increase of ≥ 7 stools per day over baseline; hospitalization indicated; limiting self-care ADL Grade 4: Life-threatening consequences; urgent intervention indicated	Grade 1: Symptomatic management: oral fluids, loperamide, avoid high fiber/lactose diet Grade ≥ 2: Hold ICI until recovery to grade ≤ 1; evaluate for infection; start 1–2 mg/kg/d steroids; gastroenterology consult. If no response within 3–5 d, consider adding infliximab. In refractory cases or cases with a contraindication to infliximab, vedolizumab can be used; earlier initiation of biologic therapy may lead to improved outcomes Grade 3: Discontinue ICI; consider resuming anti-PD-1 or anti-PD-L1 after resolution of toxicity Grade 4: Permanently discontinue ICI
Pneumonitis	Grade 1: Asymptomatic; radiographic changes only ground glass change, non-specific interstitial pneumonia Grade 2: Symptomatic; medical intervention indicated; limiting instrumental ADL Grade 3: Severe symptoms; limiting self-care ADL; oxygen indicated Grade 4: Life-threatening respiratory compromise; urgent intervention indicated (e.g., tracheotomy or intubation)	Grade 1: Consider holding ICI, reassess in 1–2 weeks, consider CT Chest Grade 2: Hold ICI; consider pulmonary consultation; consider empiric antibiotics; initiate 1–2 mg/kg/d steroids. If no improvement after 48–72 h of steroids, treat as grade 3 Grade 3–4: Permanently discontinue ICI; pulmonary and infectious disease consultation; consider empiric antibiotics; initiate 1–2 mg/kg/d steroids; assess steroid response within 48 h and plan taper over 6 weeks or longer. If no improvement after 48 h, consider adding infliximab, IVIG or mycophenolate mofetil
Rare IRAEs		
Neurologic	Grade 1: Mild symptoms Grade 2: Moderate symptoms; limiting instrumental ADL Grade 3: Severe symptoms, limiting self-care ADL Grade 4: Life-threatening consequences; urgent intervention indicated	Grade 1: Consider holding ICI Grade 2: Hold ICI, permanently discontinue if moderate encephalitis Grade 3–4: Permanently discontinue ICI Permanently discontinue ICI if: Guillain-Barre Syndrome, Transverse Myelitis, or Myasthenia Gravis Consider steroids according to guidelines, IVIG, plasmapheresis, or IV acyclovir Gabapentin, pregabalin or duloxetine for pain
Cardiovascular	Grade 1: Asymptomatic; abnormal cardiac biomarker testing, including abnormal ECG Grade 2: Abnormal screening tests with mild symptoms Grade 3: Moderately abnormal testing or symptoms with mild activity, intervention indicated Grade 4: Moderate to severe decompensation, intravenous medication or intervention required, life-threatening conditions	Grade 1: Mild abnormalities should be observed closely during therapy Grade 2: Control cardiac disease and cardiac disease risk factors Grade 3: Consider withholding ICI Grade 4: Permanently discontinue ICI Myocarditis/Pericarditis: Permanently discontinue ICI Initiate 1 mg/kg/d steroids. If no improvement within 24 h, consider adding other immunosuppressive and/or immunomodulatory agents
Rheumatologic	Grade 1: Mild pain with inflammatory symptoms, erythema, or joint swelling Grade 2: Moderate pain associated with signs of inflammation, erythema, or joint swelling; limiting instrumental ADL Grade 3: Severe pain associated with signs of inflammation, erythema, or joint swelling; irreversible joint damage; disabling; limiting self-care ADL	Mild: Supportive care, continue ICI. Consider holding ICI if mild myositis Moderate/severe: Hold ICI, administer corticosteroids For steroid-refractory cases, consider adding other immunosuppressive and/or immunomodulatory agents
Renal	Grade 1: cre 1.5–2× above baseline Grade 2: cre 2–3× above baseline Grade 3: cre > 3× above baseline or 4.0 mg/dL Grade 4: Life-threatening consequences; dialysis indicated	Grade 1: Supportive care, consider holding ICI Grade 2: Hold ICI, consider renal biopsy if feasible prior to starting steroids, nephrology consultation, initiate 0.5–1 mg/kg/d steroids for persistent grade 1–2 beyond 1 week, increase steroid to 1–2 mg/kg/d. Upon improvement to grade 1, start to taper corticosteroids Grade 3–4: Hold ICI, consider renal biopsy if feasible prior to starting steroids, nephrology consultation, start 1–2 mg/kg/d steroids For steroid-refractory cases, consider adding other immunosuppressive and/or immunomodulatory agents
Ocular	Grade 1: Asymptomatic; clinical or diagnostic observations only Grade 2: Symptomatic, limiting instrumental ADL; moderate decrease in visual acuity; anterior uveitis Grade 3; Symptomatic, limiting self- care ADL; marked decrease in visual acuity; posterior or pan-uveitis Grade 4: Blindness	Ophthalmology evaluation and vision tests (visual acuity, color vision, red reflex, fundoscopic examination, pupil size, shape, and reactivity) Grade 1: Continue ICI or hold to observe for worsening uveitis Grade 2: Hold ICI, coordinate treatment with ophthalmologist Grade 3–4: Permanently discontinue ICI, administer ophthalmic or systemic corticosteroids For steroid-refractory cases, consider adding infliximab or methotrexate for pan-uveitis

*ADL* activities of daily living; *ALT* alanine transaminase; *AST* aspartate transaminase; *BSA* body surface area; cre, creatinine; *CT* computed tomography; *CTCAE* Common Terminology Criteria for Adverse Events; *d* day; *ICI* immune checkpoint inhibitor; *IVIG* intravenous immunoglobulin; *LFT* liver function test; *SJS/TEN* Stevens-Johnson syndrome or toxic epidermal necrolysis; *TSH* thyroid-stimulating hormone; *ULN* upper limit of normal

In general, ICI treatment can be continued with close monitoring for grade 1 IRAEs, with the exception of some neurologic, hematologic and cardiac toxicities. For patients who experience grade 2 IRAEs, steroid treatment with 0.5–1 mg/kg prednisone/equivalent is recommended and ICI therapy should be held until toxicity resolves to Grade ≤ 1. For Grade 3 IRAEs, ICI treatment should be held, and dose adjustment is not recommended. Cautious retreatment with ICIs can be considered when IRAEs revert to Grade ≤ 1. Patients should be treated with high dose steroids (oral prednisolone 1–2 mg/kg/day or IV equivalent) until resolution to ≤ Grade 1, at which point steroid treatment should taper slowly over 4–6 weeks. In some cases, longer steroid tapers (6–8 weeks or more) may be required to prevent recurrent IRAEs, especially with pneumonitis and hepatitis. If there is no improvement with steroids in 1–3 days, other immunosuppressant and immunomodulatory agents can be considered. Among 5363 patients that received ICI therapy between 2013 and 2020, 6.8% required additional immunosuppressant agents for IRAEs [56]. All patients who experience grade 4 IRAEs should permanently discontinue ICI therapy, with the exception of patients with grade 4 endocrinopathies controlled by hormone replacement. Patient with hypothyroidism and asymptomatic thyrotoxicosis can continue ICI treatment. For patients with adrenal insufficiency who are hemodynamically unstable, it is recommended to hold ICI therapy until acute symptoms resolve and hormone replacement therapy is initiated [22]. Treatment for most IRAEs is typically limited to a few months, but management of thyroid disorders and adrenal insufficiency with hormone replacement therapy is generally expected to be lifelong even though it is unknown whether lifelong hormone replacement therapy is necessary. Although thyroid and adrenal disorders are managed with hormone replacement therapy and not steroid therapy, we do not know whether steroids could be useful in reversing the inflammatory process.

## Immune checkpoint inhibitor-related fatal AEs

In rare cases, serious adverse events observed with ICI therapy have been fatal. Life-threatening IRAEs reported in previous clinical trials include severe colitis, pneumonitis, encephalitis, toxic epidermal necrolysis, myocarditis, adrenal insufficiency and autoimmune type 1 DM presenting as diabetic ketosis in patients with breast cancer. ICI-related deaths may be increased with delayed diagnosis and treatment. Additionally, complications secondary to the primary adverse event can cause other severe symptoms potentially leading to death, such as infection with immune-related colitis. In a meta-analysis of patients treated with ICIs, the incidence of death from IRAEs differed based on the immunotherapy agent used; fatality rates were 0.36% with anti-PD-1, 0.38% with anti-PD-L1, 1.08% with anti–CTLA-4, and 1.23% with combined anti–PD-1/anti–PD-L1 and CTLA-4 therapy. Another meta-analysis, which included 46 studies and a total of 12,808 patients treated with anti-PD-1/anti-PD-L1, reported a fatality rate of 0.17% [50]. Incidence of death due to IRAEs also varied across tumor types. In a meta-analysis investigating 3713 patients treated with pembrolizumab, fatality rates from IRAEs in breast cancer and melanoma were 3.1% and 0.2%, respectively [51]. Fatal IRAEs varied for different treatment regimens. The most common fatal IRAE with anti–CTLA-4 therapy was colitis (70%), whereas pneumonitis (35%), hepatitis (22%), and neurotoxicity (15%) were most often seen with anti–PD-1/anti–PD-L1 therapy [57]. Fatal IRAEs reported in patients with breast cancer are summarized in Table 4.

**Table 4:** Fatal IRAEs reported in clinical trials in patients with breast cancer

Trial	Agent	Number	Fatal adverse events
Trials of immunotherapy in neoadjuvant setting			
KEYNOTE-522 [6, 30]	Pembro + CT	5	TRAEs: Sepsis, MODS, MI (*n* = 1), pneumonitis (*n* = 2) 2 patients died due to IRAEs: Adrenal crisis (n = 1), autoimmune encephalitis (*n* = 1)
I-SPY2 [29]	Pembro + CT	NR	–
IMpassion031 [7]	Atezo + CT	NR	–
GeparNuevo [42]	Durva + CT	NR	–
Trials of immunotherapy in advanced and/or metastatic setting			
KEYNOTE-012 [2]	Pembro	1	Grade 4 decreased blood fibrinogen, DIC
KEYNOTE-086 Coh A [31]	Pembro	NR	–
KEYNOTE-086 Coh B [38]	Pembro	NR	–
KEYNOTE-119 [44]	Pembro	1	Circulatory collapse
KEYNOTE-355 [5]	Pembro + CT	2	TRAEs: Acute kidney injury (*n* = 1), pneumonia (*n* = 1) No patients died due to IRAEs
NCT01375842 [25]	Atezo	2	Pulmonary HT (*n* = 1), not otherwise specified in a hospitalized patient (n = 1)
IMpassion130 [4, 26]	Atezo + CT	3	TRAEs: Autoimmune hepatitis (*n* = 1), mucosal inflammation (*n* = 1), septic shock (*n* = 1) No patients died due to IRAEs
IMpassion131 [37]	Atezo + CT	1	Polymyositis (*n* = 1)
JAVELIN [45]	Avelumab	2	TRAEs: Acute liver failure (*n* = 1), respiratory distress (*n* = 1) No patients died due to IRAEs

*Atezo* atezolizumab; *Coh* cohort; *CT* chemotherapy; *DIC* disseminated intravascular coagulation; *Durva* durvalumab; *MI* myocardial infarction; *MODS* multiple organ dysfunction syndrome; *n* number; *NR* not reported; *Pembro* pembrolizumab; *TRAEs* treatment-related adverse events

## Safety of retreatment with immunotherapy after immune-related toxicity

One of the most important issues in clinical practice is the safety of retreatment with immunotherapy following immune-related toxicity. In prospective clinical trials, ICI therapy must be permanently discontinued if a serious IRAE occurs due to study therapy. In a retrospective study, anti-PD-1 therapy was given after a severe ipilimumab-related adverse events requiring immunosuppression in patients with melanoma. Subsequent anti-PD-1 therapy resulted in a low rate of IRAE recurrence (3%), suggesting that treatment with another immunotherapy agent may be a suitable retreatment approach. Nevertheless, new IRAEs occurred frequently (34%), and many of these were high grade (21% grade 3–4) [58]. Another retrospective study explored patients with non-small cell lung cancer treated with anti–PD-1 or anti–PD-L1 therapy who experienced IRAEs requiring ICI treatment delay, treatment with glucocorticoids, or both, and who were later retreated with anti–PD-1 or anti–PD-L1 therapy. Among 39 patients who were retreated, 50% had no further IRAEs, 26% had a recurrence of the initial event, and 23% had a new event [59]. Dolladille et al. evaluated the rate of IRAE recurrence after the resumption of ICI therapy in patients with cancer. A total of 24,079 IRAEs associated with at least one ICI were identified from a database. The recurrence rate of the same IRAE was 28.8%, while a different IRAE occurred in 4.4% of patients. This study showed that colitis, pneumonitis and hepatitis were associated with a higher recurrence rate, whereas adrenal events were associated with a lower recurrence rate [60]. These results suggest that restarting ICB therapy after the resolution of IRAEs may lead to recurrent or new IRAEs. Resuming ICI therapy could be considered for select patients with appropriate monitoring and the use of standard treatment algorithms to identify and treat adverse events [60].

When restarting ICB treatment, clinicians must consider the severity of the prior event, the availability of alternative treatment options, and the overall status of the cancer. Retreatment with ICB is contraindicated if there is a life-threatening toxicity, particularly cardiac, pulmonary, or neurologic toxicity [1, 61]. The type of toxicity is also an important consideration in making retreatment decisions. In cases of thyroid, adrenal and pituitary disorders, for instance, patients can be retreated with ICI after hormone repletion and resolution of acute symptoms. Given the complexity of retreatment decisions, referral to multidisciplinary boards, including oncologists, internal medicine specialists, and organ specialists, is necessary. Recommendations for retreatment with ICIs after the occurrence of IRAEs are summarized in Table 5.

**Table 5:** Recommendations for retreatment with ICIs after IRAE occurrence

Toxicity	Cautiously retreat	Permanently discontinue
Endocrine	Hormone repletion and resolution of acute symptoms	Symptomatic pituitary inflammation
Dermatologic	Grade ≤ 1 rash or pruritus	Grade 4, Grade 3–4 bullous dermatitis, SJN/TEN
Hepatitis	Grade ≤ 2 transaminitis, bilirubin < ULN, steroid < 10 mg/d	Grade 3–4 hepatitis
Colitis	Grade 2–3 colitis, steroid < 10 mg/d	Grade 4 colitis
Pneumonitis	Grade 1–2, steroids discontinued	Grade 3–4 pneumonitis, no improvement after 48–72 h of steroids
Neurologic	Grade 1–2 peripheral neuropathy	Guillain-Barre Syndrome, moderate/severe encephalitis, transverse myelitis, Grade 2–4 myasthenia gravis,
Cardiovascular	Grade ≤ 1 myocarditis	Grade 2–4 myocarditis, pericarditis
Rheumatologic	Stabilization after adequate supportive care	Severe inflammatory arthritis that impairs ADLs
Renal	Grade 1–2, steroid < 10 mg/d	Grade 3–4 proteinuria
Ocular	Grade ≤ 2	Grade 3–4 uveitis or episcleritis

*ADL* activities of daily living; *d* day; *ICI* immune checkpoint inhibitor; *IRAEs* immune-related adverse events

## Conclusion

ICI therapy has demonstrated improving outcomes in patients with TNBC, particularly in the metastatic setting. However, with the increased use of ICIs in patients with breast cancer, clinicians have observed common and also rare IRAEs. Therefore, having an awareness of these toxicities, which differ from classical chemotherapy-related toxicities, can help lead to early recognition and optimal management. To educate patients about IRAEs and to establish physician knowledge of successful IRAE treatment strategies are important in preventing complications from potentially life-threatening IRAEs. Ongoing studies in patients with breast cancer are needed to understand the mechanisms and management of IRAEs and to prevent significant morbidity and potential mortality secondary to these agents.
